# The Miller Hospital

**Published:** 1902-08-16

**Authors:** 


### THE MILLER HOSPITAL.

### THE YEAR'S WORK.

The number of in-patients in 1901 was 352, whereas in the previous year it was 309. Accidents and casualties and dental patients also showed an increase (there were 7,089 of the former, and 1,724 of the latter, compared with 6,252

and 1,612 respectively), while of out-patients, home, and lying-in patients, there was a slight decrease in numbers. The "estimated attendances" were 73,342, and in 1900, 16,841. Additional accommodation, says the report, is imperative. There is urgent need for more beds, and the income to maintain them. Improved accommodation is wanted for the staff, and larger rooms for the ophthalmic department, which has become a most important branch, and the pressing need for improvement in the out-patients' department has been strongly urged by the council of King Edward's Fund. At present, the out-patient and ophthalmic work is carried on in an iron building, which it is hoped to replace by a permanent extension.

The total ordinary income was £3,688; ordinary expenditure, £3,721. There were legacies of £180, and £168 was invested in India 3 per cent. stock. This was a life subscription. King Edward's Fund gave a donation of £500, the Sunday Fund £239 10s., and the Saturday Fund £73 13s. Dividends on invested property amounted to £355.

